# Investigation of the mechanism of tanshinone IIA to improve cognitive function via synaptic plasticity in epileptic rats

**DOI:** 10.1080/13880209.2022.2157843

**Published:** 2022-12-22

**Authors:** Chen Jia, Rui Zhang, Liming Wei, Jiao Xie, Suqin Zhou, Wen Yin, Xi Hua, Nan Xiao, Meile Ma, Haisheng Jiao

**Affiliations:** a Department of Pharmacy, Lanzhou University Second Hospital, Lanzhou, China; b Department of Pharmacy, Gansu Provincial Maternity and Child-care Hospital, Lanzhou, China; c Department of Pharmacy, The Second Affiliated Hospital of Xi’an Jiaotong University, Xi’an, China; d College of Pharmacy, Lanzhou University, Lanzhou, China

**Keywords:** Cognitive impairment, epilepsy, SYN, PSD-95, mossy fibre sprouting

## Abstract

**Context:**

Tanshinone IIA is an extract of *Salvia miltiorrhiza* Bunge (Labiatae) used to treat cardiovascular disorders. It shows potential anticonvulsant and cognition-protective properties.

**Objective:**

We investigated the mechanism of tanshinone IIA on antiepileptic and cognition-protective effects in the model of epileptic rats.

**Materials and methods:**

Lithium chloride (LiCl)-pilocarpine-induced epileptic Wistar rats were randomly assigned to the following groups (*n* = 12): control (blank), model, sodium valproate (VPA, 189 mg/kg/d, positive control), tanshinone IIA low dose (TS IIA-L, 10 mg/kg/d), medium dose (TS IIA-M, 20 mg/kg/d) and high dose (TS IIA-H, 30 mg/kg/d). Then, epileptic behavioural observations, Morris water maze test, Timm staining, transmission electron microscopy, immunofluorescence staining, western blotting and RT-qPCR were measured.

**Results:**

Compared with the model group, tanshinone IIA reduced the frequency and severity of seizures, improved cognitive impairment, and inhibited hippocampal mossy fibre sprouting score (TS IIA-M 1.50 ± 0.22, TS IIA-H 1.17 ± 0.31 vs. model 2.83 ± 0.31), as well as improved the ultrastructural disorder. Tanshinone IIA increased levels of synapse-associated proteins synaptophysin (SYN) and postsynaptic dense substance 95 (PSD-95) (SYN: TS IIA 28.82 ± 2.51, 33.18 ± 2.89, 37.29 ± 1.69 vs. model 20.23 ± 3.96; PSD-95: TS IIA 23.10 ± 0.91, 26.82 ± 1.41, 27.00 ± 0.80 vs. model 18.28 ± 1.01).

**Discussion and conclusions:**

Tanshinone IIA shows antiepileptic and cognitive function-improving effects, primarily via regulating synaptic plasticity. This research generates a theoretical foundation for future research on potential clinical applications for tanshinone IIA.

## Introduction

Epilepsy has a high prevalence as a common neurological disorder in clinical practice. Cognitive impairment is the most common complication of epilepsy and is a determinant of reduced quality of survival in patients with epilepsy (Lancman et al. 2016; Ives-Deliperi and Butler 2021). Recent studies have shown that cognitive impairment usually appears in the early stages of epilepsy onset (Reyes et al. 2021). Most newly diagnosed epilepsy patients have a cognitive impairment, ranging from mild to severe, particularly visual and spatial impairment (Jackson-Tarlton et al. 2020). Traditional and newer antiepileptic drugs (AEDs), such as sodium valproate (VPA, a broad-spectrum AED), carbamazepine and topiramate, are effective in controlling most seizures. Still, these AEDs do not improve cognitive function. As epilepsy is in remission, cognitive impairment persists or worsens (Choo et al. 2019; Quon et al. 2020). Improving cognitive deficits in epilepsy patients is currently the focus of AED research.

Traditional Chinese medicine has been treating epilepsy for hundreds of years. The properties of herbal medicines, such as relatively low toxicity and overall modulating effects, may provide new approaches and ideas as potential treatment drugs for better epilepsy control and may improve cognitive impairment in epileptic patients. Many herbal medicines and monomers, such as *Glycyrrhiza glabra* L. (Fabaceae), honokiol and salvianolic acid B, have been found to have antiepileptic properties in recent years (Yu et al. 2019; El-Saber et al. 2020; Li et al. 2020). The herbal medicine *Salvia miltiorrhiza* Bunge (Labiatae) has the effects of activating blood circulation, cooling blood, subduing swelling, removing irritation and clearing the heart. Our previous study found that its compound preparation, Compound Danshen dripping pills had synergistic antiepileptic effects and improved the cognitive function of epileptic rats (Jia et al. 2018). The pharmacologically active substance tanshinone IIA is isolated from *Salvia miltiorrhiza* and is extensively used in clinical practice to prevent and alleviate various diseases, including cardiovascular diseases, cancer and liver fibrosis. According to recent research, tanshinone IIA also has a significant pharmacological effect on regulating neurological functions. In Alzheimer’s disease (AD) study, Ding et al. (2020) found that tanshinone IIA could improve cognitive impairment by repressing neuroinflammatory responses in mice. In another study, He et al. (2020) found that tanshinone IIA could improve cognitive impairment by suppressing endoplasmic reticulum stress-induced apoptosis. A study of acellular neurotoxicity showed that tanshinone IIA might benefit the nervous system by protecting mitochondria and promoting cell autophagy (Cheng et al. 2019).

Learning and memory are molecular processes governed by synaptic plasticity, which closely correlates with many cognitive dysfunctional diseases, such as AD and epilepsy-induced cognitive dysfunction (Göl et al. 2019; Humeau and Choquet 2019; Cuestas Torres and Cardenas 2020). Synaptic remodelling aids in the formation of the nervous system in the brain and regulatory processes following injury, and it is closely linked to the repair of cognitive dysfunction (Sgobio et al. 2010). Therefore, improving synaptic plasticity is beneficial in alleviating cognitive function decline (Bonansco and Fuenzalida 2016). Learning and memory may be influenced by different synaptic plasticity changes that regulate synaptic transmission, but the mechanisms by which synaptic plasticity changes are independently regulated in synapses remain unclear (Humeau and Choquet 2019; Mansvelder et al. 2019).

To investigate tanshinone IIA's effects on epileptic seizures and cognitive function improvement, as well as its mechanism of action, we established an epilepsy model in rats by injecting lithium chloride and pilocarpine (LiCl–pilocarpine) intraperitoneally. This study intends to advance new ideas and a theoretical framework for investigating novel tanshinone IIA clinical indications and preventing and treating cognitive dysfunction in epilepsy.

## Materials and methods

### Animals and grouping

Laboratory Animal Service Centre of Lanzhou University provided clean male Wistar rats weighing 200 ± 20 g (license number SCXK (G) 2018-0002). Group-housed experimental rats (22 ± 3 °C, 40–55% humidity, 12 h light/dark cycle) were given a standard diet and access to water *ad libitum*. The experimental animal procedures and sample collections were performed following the animal care guidelines of the National Institutes for Health and approved by the Animal Ethics Committee of Lanzhou University Second Hospital (approval number: D2021-051).

Male Wistar rats were randomly divided into six groups (*n* = 12): control (blank), model, VPA (positive control), tanshinone IIA low dose (TS IIA-L), medium dose (TS IIA-M) and high dose (TS IIA-H) groups. Except for the control group, the rats in each group were injected intraperitoneally with LiCl (3 mmol/kg, #608D023; Solarbio Science & Technology Co., Ltd., Beijing, China) and then injected with atropine sulphate (1 mg/kg, #H41021256; Tianjin Pharma. Co., Ltd., Tianjin, China) after 18 h. The epilepsy model was established by injecting pilocarpine (35 mg/kg, #P6503; Sigma-Aldrich, St. Louis, MO) after 0.5 h. The rats in the control group received the same treatment with LiCl and atropine sulphate but received an equivalent amount of normal saline as a replacement for pilocarpine (Zhou et al. 2022). If seizures were not induced within 0.5 h, pilocarpine 10 mg/kg was administered every 30 min until a continuous state of epilepsy was established. Pilocarpine’s maximum dose was 60 mg/kg. After 1 h of status epilepticus (SE), the seizure was terminated by intraperitoneal injection of 10% chloral hydrate (10 mg/kg) (Contreras-García et al. 2018; Li et al. 2019). Epileptic seizures were assessed by Racine stages (Phelan et al. 2015) as follows: 0 = normal, no reaction, 1 = mouth and facial movements, 2 = chewing and head nodding or severe facial clonus, 3 = forelimb clonus, 4 = forelimb convulsion and rearing and 5 = forelimb clonus, rearing and falling. Only stage 4 or higher rats were chosen for subsequent drug administration, behavioural observation, Morris water maze (MWM) trials and mechanism research experiments.

### Drug administration

**Figure 1.**
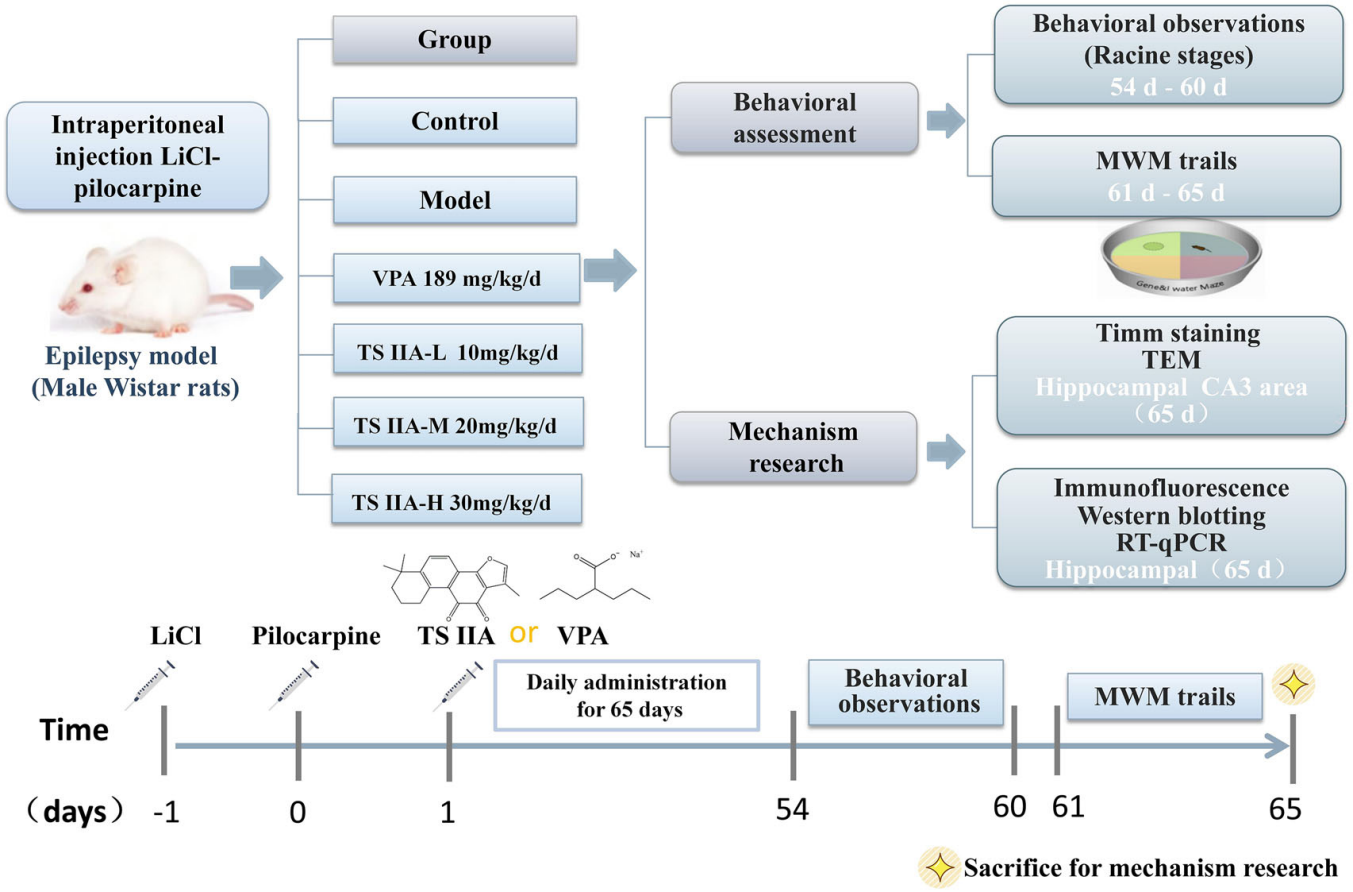
Flowchart and timetable for the experiment. The LiCl–pilocarpine epilepsy model was established from day –1 to 0; tanshinone and valproate were administered from day 1 to 65; behavioural observation was performed from day 54 to 60; MWM trials were conducted from day 61 to 65. Finally, on day 65, the rats were sacrificed for mechanism research experiments. TS IIA: tanshinone IIA; VPA: sodium valproate; MWM trails: Morris water maze (MWM) trails; TEM: transmission electron microscopy.

Tanshinone IIA (#S31459; Shanghai Yuanye Biotech, Shanghai, China) was dissolved in 0.5% carboxymethylcellulose sodium (CMC-Na) solution and then diluted to various concentrations in normal saline. VPA (0.1 g/pill, #181035; Hunan Xiangzhong Pharm. Co., Ltd., Hunan, China) was dissolved in normal saline to get a 40 mg/mL concentration. The rats in the TS IIA-L, TS IIA-M and TS IIA-H groups underwent gavage with 10, 20 and 30 mg/kg tanshinone IIA once daily for 65 consecutive days. The rats in the VPA group were given 189 mg/kg VPA by gavage once a day for 65 consecutive days. The dose was determined according to the Meeh-Rubner formula by conversion of body surface area and literature reference dose (Gouma et al. 2012; He et al. 2020). The control and the model groups were administered the same volume of normal saline by gavage, and subsequent experiments were performed after continuous gavage for 60 days. The flow chart and timetable for the investigation are revealed in Figure 1.

### Behavioural observations

Rat seizures in each group were observed daily from 9:00 am to 10:00 am using Racine stages as a reference. After 53 days of gavage administration, the seizure grade and the frequency of spontaneous seizures above grade III were recorded continuously for one week in each group of rats.

### MWM trials

The experimental equipment includes a circular pool and an automatic video analysis system (ZS-001; Beijing Zhongshi Technology Co., Ltd., Beijing, China). The circular collection was divided into four quadrants (W, E, N, S) with a fixed entry point in each quadrant. The pool was filled with sufficient water, kept at a constant temperature of 22 °C, with a white platform in the middle of the N quadrant, and stirred with titanium dioxide until the pool was opaque. There were two parts to the experiment: positioning navigation and space exploration. The positioning navigation experiment lasted 4 d and was tested four times daily. Each test involved placing rats face the pool wall from different quadrants and measuring how long they took to climb onto the platform as the escape latency. The experiment on spatial exploration without the platform began the day after the positional navigation trial ended. From the S quadrant (the quadrant farthest from the original medium), rats were placed into the water, and the platform-crossing frequency within 120 s was recorded (Vorhees and Williams 2006).

### Timm staining experiment

Morphological alteration and mossy fibre sprouting (MFS) were evaluated using Timm staining (Wang et al. 2021). The rats were given an intraperitoneal injection of 10% chloral hydrate (350 mg/kg), followed by transcardial infusions of normal saline, 0.4% Na_2_S solution, and 4% paraformaldehyde (PFA). Brains were immediately severed and preserved for 48 h in 4% PFA. Brain tissues were immersed in a 20–30% sucrose solution until they sank, and frozen slices were produced and stored at −20 °C for subsequent experiments. After 30 min of drying at room temperature, sections were immersed in Timm staining solution in a dark room and stained at 37 °C for 2 h. After dehydration by an ethanol gradient and clearing in xylene, the sections were sealed with neutral resin, observed under a light microscope (BH-2; Olympus, Tokyo, Japan), photographed, and recorded for histopathological changes in the rats’ hippocampi. The pathological changes in the abovementioned groups of sections were evaluated concerning the Gregory MFS criteria (Holmes et al. 1999).

### Transmission electron microscopy experiments

Transcardial perfusion of normal saline followed by 4% PFA was given to the rats after deep anaesthesia. When the rats developed convulsions and stiffness in their limbs, the brains were quickly decapitated, and hippocampi were separated on ice. According to the mapping map of the brain area, the tissues in the CA3 region of the hippocampi were taken and dissected into 1 mm^3^ pieces or 1 mm^2^ strips. The tissues were quickly immersed in a 2.5% glutaraldehyde fixative solution and fixed overnight at 4 °C. The hippocampi were rinsed with 0.1 M phosphate-buffered saline (PBS), set in 1% osmic acid at 4 °C for 2 h, and then rinsed again with PBS the following day. The hippocampi were sequentially placed in 50–100% acetone solution, graded for dehydration, and then embedded. The embedded tissues were subjected to ultrathin sectioning, uranyl acetate and citric acid. We photographed and recorded ultrastructural alterations in hippocampal neurons in the CA3 region of the brain using transmission electron microscopy following double labelling with lead citrate (Tecnai G2 Spirit Bio-Twin; FEI, Hillsboro, ‎OR).

### Immunofluorescence assay

Frozen sections of rats were prepared according to the Timm staining method described above. After drying at room temperature for 10 min, the slices were rinsed with PBS. The antigen was repaired with proteinase K solution (#P1120; Solarbio Science & Technology Co., Ltd., Beijing, China) for 20 min at 37 °C and permeabilized with 0.3% Triton X-100 solution (#T8200; Solarbio Science & Technology Co., Ltd., Beijing, China) for 15 min at room temperature. We used 5% bovine serum albumin (BSA) to block nonspecific binding sites for 30 min at 37 °C. At 4 °C, the slices were incubated with the following primary antibodies overnight: mouse anti-synaptophysin (SYN) (1:200, #M05049-3; Boster Biotech, Wuhan, China) and rabbit anti-postsynaptic dense substance 95 (PSD-95) (1:200, #M02128-1; Boster Biotech, Wuhan, China). Slices were rinsed with PBS and then incubated for 1 h with fluorescently labelled secondary antibodies: sheep anti-mouse Cy3 (1:200, #BA1031; Boster Biotech, Wuhan, China) and sheep anti-rabbit DyLight 488 (1:200, #BA1127; Boster Biotech, Wuhan, China) in the dark room. Following a PBS rinse, the slices were sealed with an anti-fluorescence blocker. Images were taken using a fluorescence microscope (BX-51; Olympus, Tokyo, Japan). The data were examined and counted for positive expression in the field of vision using Image-Pro Plus 6.0 software (Media Cybernetics, Bethesda, MD).

### Western blot method

The hippocampi were freshly isolated after the rats were quickly sacrificed by having their brains removed while they were under anaesthesia. A lysis buffer containing ice-cold radioimmunoprecipitation assays (RIPA) was used to homogenize the samples (#MA0151; Meilune Biotech, Dalian, China). Next, the supernatants were collected by centrifugation at 12,000 rpm for 10 min, and the total protein content was measured using a BCA protein assay kit (#PC0020; Solarbio Science & Technology Co., Ltd., Beijing, China). SDS-polyacrylamide gels were used to separate equal amounts of protein, which were then transferred to PVDF membranes (Millipore, Billerica, MA) at 110 V for 80 min. After blocking with 5% skimmed milk powder in Tris-buffered saline with Tween 20 (TBST), PVDF membranes were incubated at room temperature for 2 h and then overnight at 4 °C with the following primary antibodies: mouse anti-SYN (1:800, #M05049-3; Boster Biotech, Wuhan, China), mouse anti-PSD-95 (1:2000, #M02128-1; Boster Biotech, Wuhan, China) and rabbit anti-β-actin (1:8000, #YT672; Baiaolaibo, Beijing, China). The PVDF membranes were then incubated with HRP-conjugated goat anti-rabbit and goat anti-mouse secondary antibodies (1:8000) for 2 h at room temperature after washing in TBST. A chemiluminescence kit (ECL) was used to visualize target proteins, and Image J was used to analyse the results (NIH, Bethesda, MD). The relative band density ratio was calculated by normalizing all values against β-actin.

### Real-time quantitative polymerase chain reaction (RT-qPCR)

The rats were given anaesthesia before being sacrificed by quickly removing their brains and isolating their hippocampi. Total RNA was extracted using the TRIzol reagent. Using a microvolume UV-Vis Spectrophotometer (NANODrop 2000; Thermo Scientific, Waltham, MA), we determined the concentration and purity of the extracted RNA samples. Total mRNA (1 µg) was reverse-transcribed into cDNA following the manufacturer’s instructions using the Evo M-MLV RT Premix reagent Kit. The primers mouse SYN (forward, 5′-GGACCCTAGCAGTGAGGCTTATGA-3′; reverse, 5′-GGGCAGATCTTAGGACAGTGGGTA-3′); mouse PSD-95 (forward, 5′-ACTGCATCCTTGCGAAGCAAC-3′; reverse, 5′-CGTCAATGACATGAAGCACATCC-3′); and mouse β-actin (forward, 5′-GGAGATTACTGCCCTGGCTCCTA-3′; reverse, 5′-GACTCATCGTACTCCTGCTTGCTG-3′) were designed and synthesized by Shanghai Biotechnology Co., Ltd. (Shanghai, China). RT-qPCR was used to amplify the cDNAs (2 μL) using the above-mentioned primers. During an amplification, 95 °C was applied for 30 s, followed by 95 °C for 5 s, followed by 40 cycles of 60 °C (30 s). On a Real-Time PCR Machine (LightCycler 96; Roche, Mannheim, Germany), RT-qPCR was done according to the instructions using SYBR^®^ Green Taq HS Premix. Using the 2^−ΔΔCt^ method with β-actin as the internal control, we calculated the relative level of expression of the target gene.

### Data analysis

The experimental data were analysed by SPSS Statistics (version 25.0, IBM Corp., Armonk, NY), and we expressed the measurement data as $\bar{x}$ ± SEM (standard error of the mean). One-way analysis of variance (ANOVA) was used to assess the experimental results, and the LSD test was used to compare the two groups. Repeated-measures ANOVA was performed on the results of the water maze experiment. Statistical differences were indicated by *p* < 0.05, and *p* < 0.01 revealed statistically significant differences.

## Results

### Effects of tanshinone IIA on the degree of seizure

**Figure 2.**
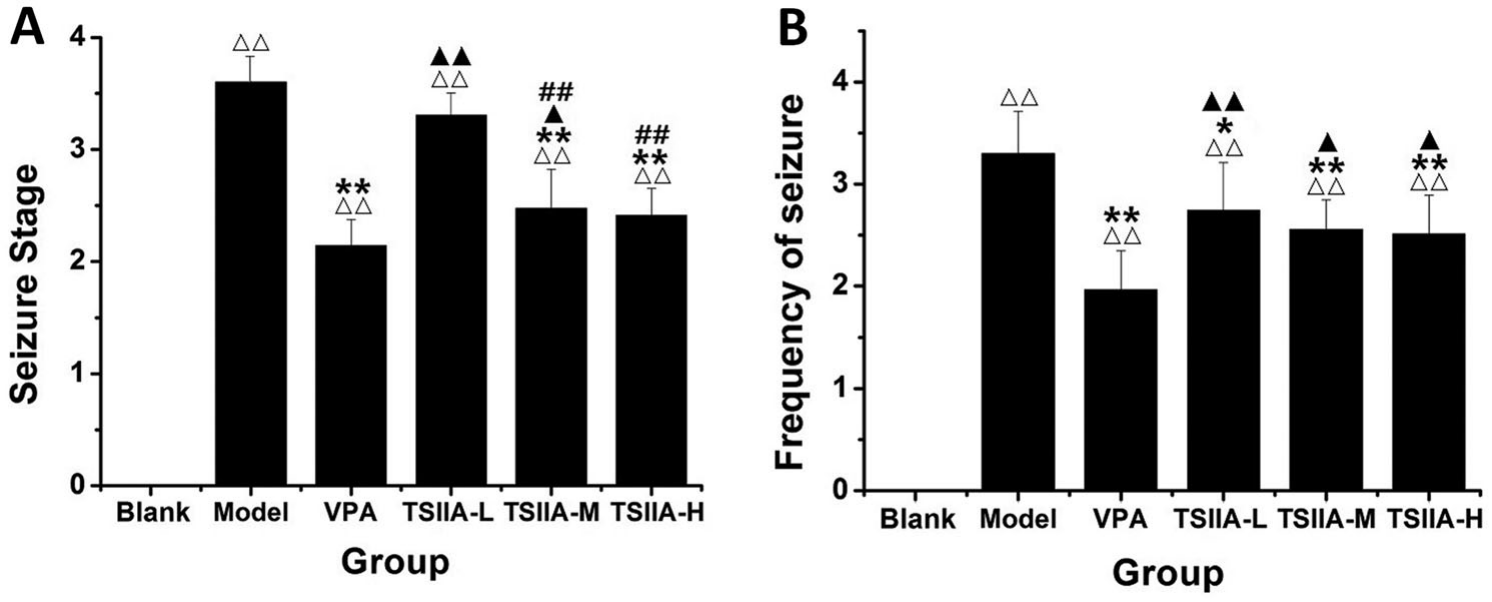
Effects of tanshinone IIA on the degree of seizure in LiCl–pilocarpine induced mice. (A) Stages of epileptic seizures; results of seizure severity and frequency. (B) Frequency of spontaneous recurrent seizures (SRS). SRS were recorded three times a day for one week. According to Racine stages, only the seizures of stage 3 or higher were recorded. The results are presented as mean ± SEM. ^ΔΔ^*p* < 0.01 vs. control; *p < 0.05, **p < 0.01 vs. model; ^▲^*p* < 0.05, ^▲▲^*p* < 0.01 vs. VPA; ^##^*p* < 0.01 vs. TS IIA-L (*n* = 9 per group).

After injecting LiCl–pilocarpine, the rats gradually developed SE, including salivation, nodding, forelimb clonus, rolling and falling. When the SE lasted more than 1 h, a 10% chloral hydrate injection was administered intraperitoneally. After the latency period, we observed chronic epilepsy in rats with grades I through V. A behavioural evaluation was done 53 days after the medication was administered. Seizures were considerably less severe and less frequent in the VPA group than in the model group (Figure 2(A)). Tanshinone IIA administration reduced the severity of seizures in rats compared to the model group, with a noticeable reduction in the severity of seizures in the TS IIA-M and TS IIA-H groups. Compared to the model group, all tanshinone IIA dose groups demonstrated a decrease in seizure frequency (Figure 2(B)). Increases in tanshinone IIA dose positively correlated with seizure severity and frequency improvements.

### Effect of tanshinone IIA on cognitive impairment

**Figure 3.**
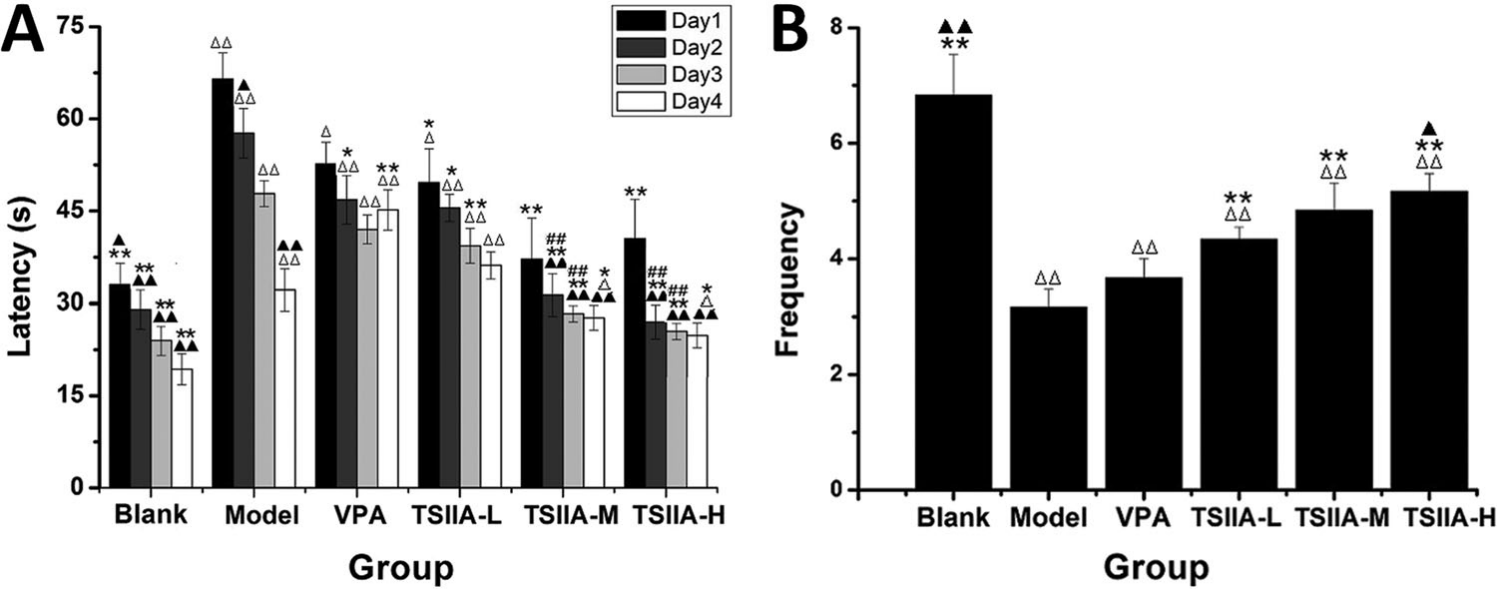
Effect of tanshinone IIA on ameliorates cognitive impairment in LiCl–pilocarpine-induced mice. (A) Average escape latencies. (B) Frequency crossing the platform. The results are presented as means ± SEM. ^Δ^*p* < 0.05, ^ΔΔ^*p* < 0.01 vs. control; *p < 0.05, **p < 0.01 vs. model; ^▲^*p* < 0.05, ^▲▲^*p* < 0.01 vs. VPA; ^##^*p* < 0.01 vs. TS IIA-L. (*n* = 10 per group).

MWM tests were conducted 60 days after tanshinone IIA administration to assess its impact on cognitive impairment. In the place navigation test, the escape latency of each group tended to decrease as training time extended, as depicted in Figure 3(A). On each training day, the model, VPA and TS IIA-L groups exhibited significantly higher escape latencies than the control group. Compared with the model group, VPA, TS IIA-L, TS IIA-M and TS IIA-H groups were all shortened for escape latency. Only the TS IIA-M and TS IIA-H groups presented a statistically significant reduction in escape latency over the VPA group. The frequency of platform crossings was considerably reduced in all experimental groups compared to the control group in the spatial probe trials (Figure 3(B)). Platform crossing frequency was significantly higher in the TS IIA-L, TS IIA-M and TS IIA-H groups than in the model group. As a result, only the frequency of platform crossings among TS IIA-M and TS IIA-H groups was substantially higher than that of the VPA group. Crossing frequency increased with increasing doses of tanshinone IIA and was higher in the TS IIA-H group compared to the TS IIA-L group. According to the findings, tanshinone IIA could improve learning and memory in epileptic rats.

### Effect of tanshinone IIA on MFS and ultrastructure damage in the hippocampus

**Figure 4.**
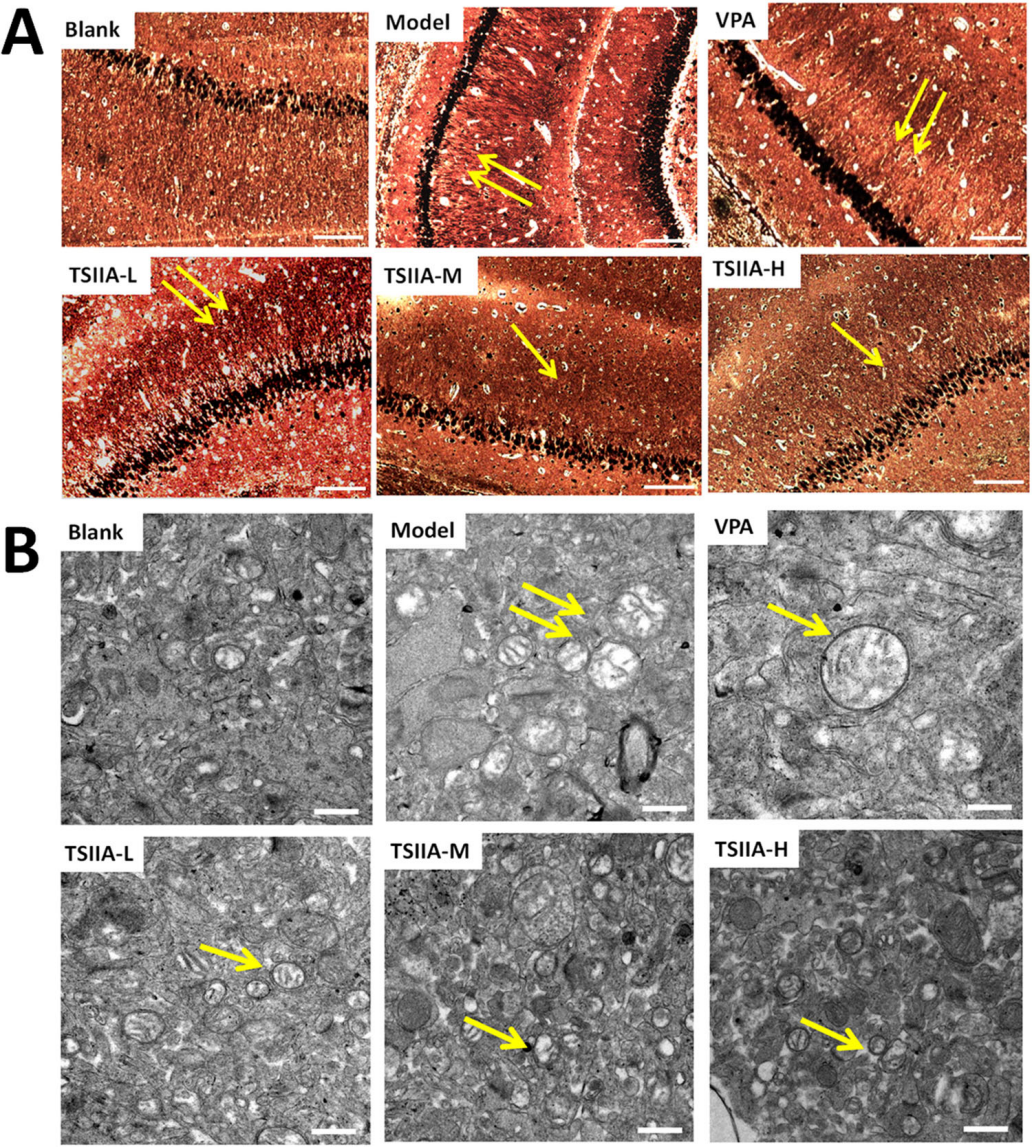
Effects of tanshinone IIA on mossy fibre sprouting (MFS) and ultrastructure deterioration in the hippocampus. (A) Timm staining was utilized to evaluate the effects of tanshinone IIA on MFS (×200, scale bar: 100 μm). Seizures increased granules in the stratum pyramidal or stratum oriens of the CA3 region, as indicated by the yellow arrows, with the number of arrows indicating the granule increase. Massive MFS induced by LiCl–pilocarpine was mitigated by tanshinone IIA treatment. (B) Transmission electron microscopy was utilized to assess ultrastructure damage (×20,000, scale bar: 0.5 μm). As indicated by the yellow arrows, seizures increased vacuolar degeneration in the CA3 region, and the number of arrows reflected the quantity and degree of vacuolar degeneration. The treatment with tanshinone IIA enhanced the ultrastructural disorder and vacuolar degeneration (*n* = 6 per group).

Seizures could lead to MFS of the hippocampal dentate gyrus and synaptic loss (Sutula et al. 1989; Lin et al. 2011). We used Timm staining and transmission electron microscopy (Figure 4, Table 1) to estimate the effects of tanshinone IIA on MFS and ultrastructure damage after the MWM test. The hippocampal CA3 MFS score was calculated using the Gregory MFS criteria (Holmes et al. 1999) as follows: 0 = no granules in the stratum pyramidal or stratum oriens; 1 = occasional granules; 2 = occasional to moderate granules; 3 = prominent granules; 4 = prominent granules occurring in the near-continuous distribution along the whole CA3 region; 5 = dense laminar band of granules. Seizures increased granules in the stratum pyramidal or stratum oriens of the CA3 region (illustrated by yellow arrows in Figure 4(A)). In contrast to the control group, the model group displayed abnormal MFS (2.83 ± 0.31) and ultrastructural disorder. Various visual field regions exhibited vacuolated degenerative structures (illustrated by the yellow arrows in Figure 4(B)), while the nuclei were fixed and the tissue shape was muddled. The morphology in the TS IIA-M and TS IIA-H groups tended to be normal without apparent structural damage. The MFS scores in the TS IIA-L (2.00 ± 0.26), TS IIA-M (1.50 ± 0.22) and TS IIA-H (1.17 ± 0.31) groups were lower than the model group (Table 1). The MFS, vacuolar degeneration and ultrastructural disorder were all abnormal in the VPA. Compared to the model group, the VPA group showed an improved hippocampal structure and less vacuolar degeneration field, but the degree of vacuolar degeneration increased. Small MFS were still discernible in the visual field for the VPA group, and there was no noticeable difference between the VPA and model groups (2.17 ± 0.17 vs. 2.83 ± 0.31). Tanshinone IIA treatment improved the disorganized ultrastructure and blurred tissue morphology of the CA3 area observed in transmission electron microscopy, implying that tanshinone IIA may ameliorate the abnormal germination of mossy fibres as well as the ultrastructural disorder and vacuolar degeneration of the hippocampal CA3 regions caused by epilepsy.

**Table 1.** Moss fibre germination (MFS) score of each group in the Timm staining.

Group	MFS (*x* ± *s*)
Blank	0.17 ± 0.17
Model	2.83 ± 0.31^ΔΔ^
VPA	2.17 ± 0.17^ΔΔ^
TSIIA-L	2.00 ± 0.26^ΔΔ,^*
TSIIA-M	1.50 ± 0.22^ΔΔ,^**
TSIIA-H	1.17 ± 0.31^ΔΔ,^**^,▲▲,#^

Results are presented as mean ± SEM.

^ΔΔ^*p* < 0.01 vs. blank; *p < 0.05, **p < 0.01 vs. model; ^▲▲^*p* < 0.01 vs. VPA; ^#^*p* < 0.05 vs. TS IIA-L (*n* = 6 per group).

### Immunofluorescence study of tanshinone IIA's effect on hippocampus synaptophysin and postsynaptic dense substance 95 expression

**Figure 5.**
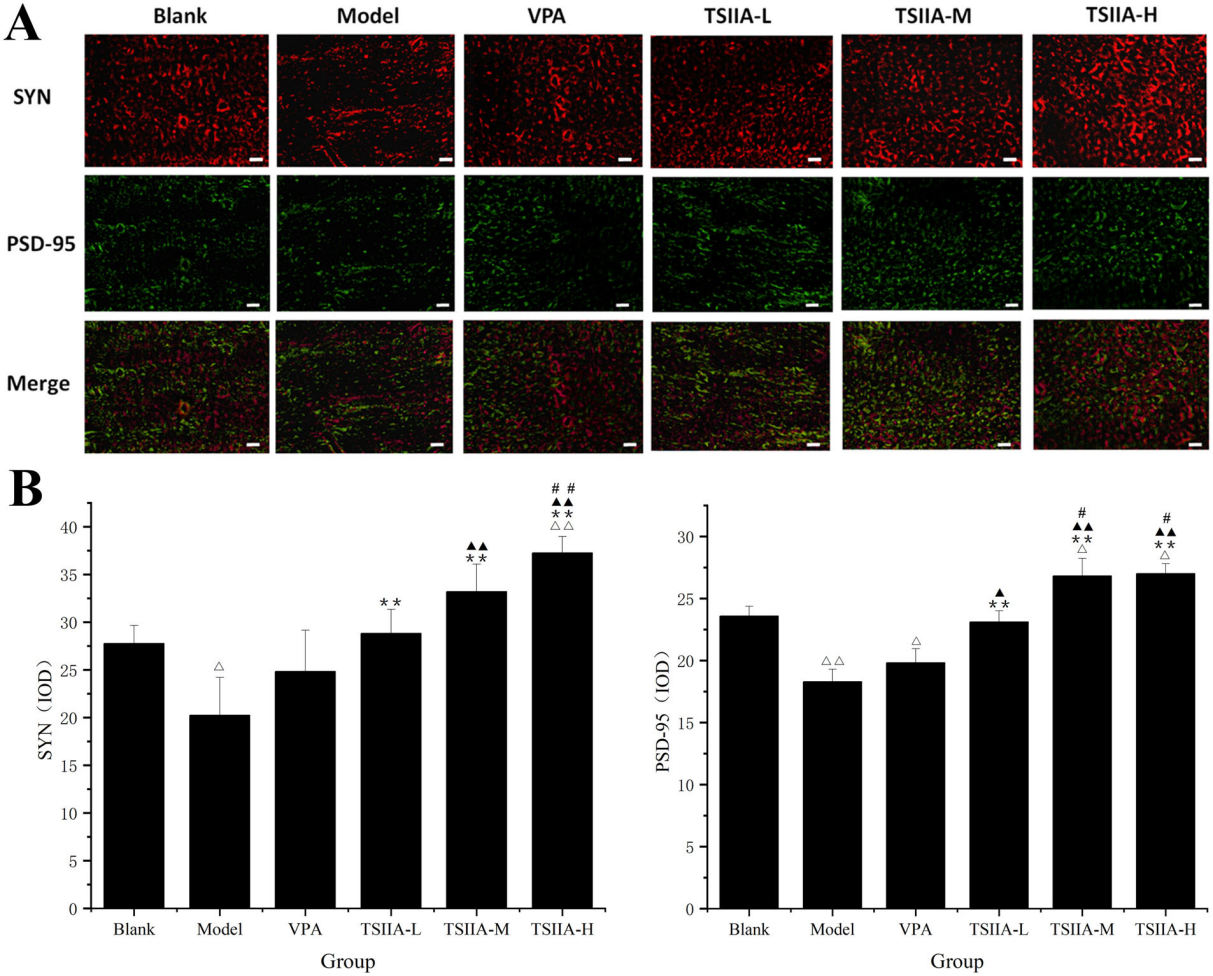
Immunofluorescence study of tanshinone IIA's effect on hippocampus SYN (red) and PSD-95 (green) expression. (A) Single and double fluorescence staining (×400, scale bar: 50 μm). (B) Immunofluorescence expression results of SYN and PSD-95 were counted and analysed with Image-Pro Plus 6.0 software. Results are presented as mean ± SEM. ^Δ^*p* < 0.05, ^ΔΔ^*p* < 0.01 vs. blank; **p < 0.01 vs. model; ^▲^*p* < 0.05, ^▲▲^*p* < 0.01 vs. VPA; ^#^*p* < 0.05, ^##^*p* < 0.01 vs. TS IIA-L (*n* = 6 per group).

Proteins associated with synapses, especially SYN and PSD-95, play a crucial role in memory and synaptic plasticity (Luo et al. 2013; Guarnieri et al. 2017). Single and double fluorescence staining showed that tanshinone IIA could enhance the expression of SYN and PSD-95 in epileptic rats (Figure 5, Table 2). In all tanshinone IIA groups, the expression of SYN and PSD-95 was significantly greater than in the model group (SYN: TS IIA-L 28.82 ± 2.51, TS IIA-M 33.18 ± 2.89, TS IIA-H 37.29 ± 1.69 vs. model 20.23 ± 3.96; PSD-95: TS IIA-L 23.10 ± 0.91, TS IIA-M 26.82 ± 1.41, TS IIA-H 27.00 ± 0.80 vs. model 18.28 ± 1.01). In contrast, there was no significant difference between the VPA (SYN: 24.82 ± 4.33, PSD-95: 19.81 ± 1.14) and model groups (SYN: 20.23 ± 3.96, PSD-95: 18.28 ± 1.01). SYN expression was upregulated in the TS IIA-H group compared to the control group (37.29 ± 1.69 vs. 27.75 ± 1.91; Figure 5(A)). At the same time, it was similar in TS IIA-L and TS IIA-M groups with no significant difference. SYN expression was significantly lower in the model groups compared to the control group (20.23 ± 3.96 vs. 27.75 ± 1.91). In terms of PSD-95 expression, both the TS IIA-M and TS IIA-H groups showed significant increases, while the TS IIA-L group was close to the control group (23.58 ± 0.79; Figure 5(B)). Compared to the control group, an apparent decrease in PSD-95 expression was found in the model and VPA groups (control 23.58 ± 0.79 vs. model 18.28 ± 1.01, VPA 19.81 ± 1.14).

**Table 2.** Immunofluorescence expression results of SYN and PSD-95 protein in the hippocampi of each group.

Group	SYN (IOD)	PSD-95 (IOD)
Blank	27.75 ± 1.91	23.58 ± 0.79
Model	20.23 ± 3.96^Δ^	18.28 ± 1.01^ΔΔ^
VPA	24.82 ± 4.33	19.81 ± 1.14^Δ^
TSIIA-L	28.82 ± 2.51**	23.10 ± 0.91**^,▲^
TSIIA-M	33.18 ± 2.89**^,▲▲^	26.82 ± 1.41^Δ,^**^,▲▲,#^
TSIIA-H	37.26 ± 1.69^ΔΔ,**,▲▲,##^	27.00 ± 0.80^Δ,**,▲▲,#^

Results are presented as mean ± SEM.

^Δ^*p* < 0.05, ^ΔΔ^*p* < 0.01 vs. blank; **p < 0.01 vs. model; ^▲^*p* < 0.05, ^▲▲^*p* < 0.01 vs. VPA; ^#^*p* < 0.05, ^##^*p* < 0.01 vs. TS IIA-L. (*n* = 6 per group)

### Western blot study of tanshinone IIA's effect on hippocampus SYN and PSD-95 expression

**Figure 6.**
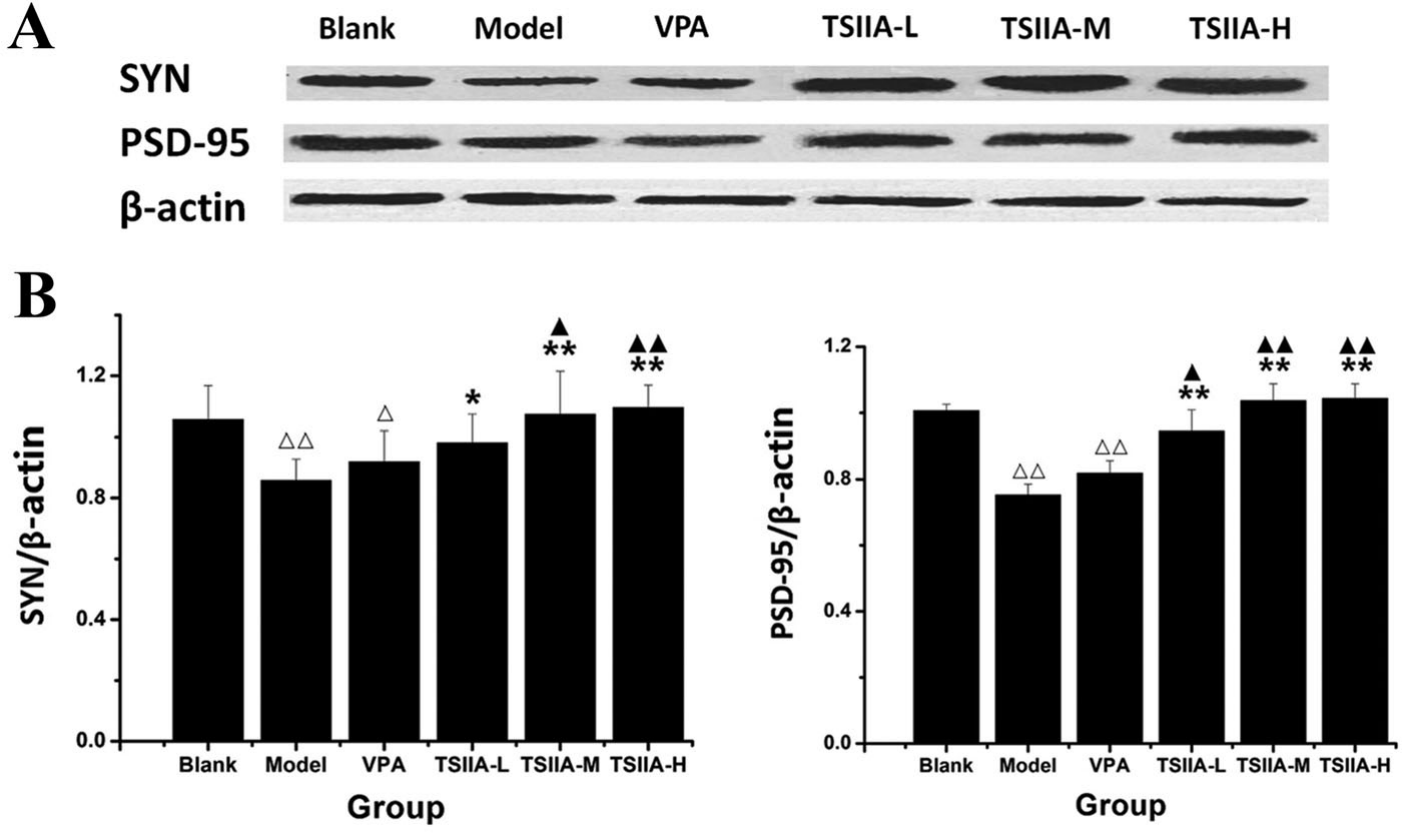
Western blot study of tanshinone IIA's effect on hippocampus SYN and PSD-95 expression. (A) Western blotting was used to evaluate the protein expression of SYN and PSD-95 in the hippocampus, and β-actin was used as an internal control. (B) Densitometry analysis was performed using the Image J software. Results are presented as means ± SEM. ^Δ^*p* < 0.05, ^ΔΔ^*p* < 0.01 vs. blank; *p < 0.05, **p < 0.01 vs. model; ^▲^*p* < 0.05, ^▲▲^*p* < 0.01 vs. VPA (*n* = 6 per group).

As compared to the model group, SYN expression increased in the TS IIA-L, TS IIA-M and TS IIA-H groups (Figure 6(A)), while PSD-95 was overexpressed in all tanshinone groups (Figure 6(B)). With an increase in tanshinone IIA dosage, the expression of SYN and PSD95 proteins appeared to rise. SYN expression was enhanced in the TS IIA-M and TS IIA-H groups compared to the control group, while PSD-95 levels were raised in all tanshinone IIA groups. SYN and PSD-95 expressions were significantly lower in the model and VPA groups compared to the control group. Our results showed that tanshinone IIA increased the expression of the synapse-associated proteins SYN and PSD-95 in the hippocampi of rats treated with LiCl–pilocarpine. The immunofluorescence assay results and the western blot test were in agreement.

### Analysis of the effects of tanshinone IIA on mRNA expression of SYN and PSD-95 in the hippocampus

**Figure 7.**
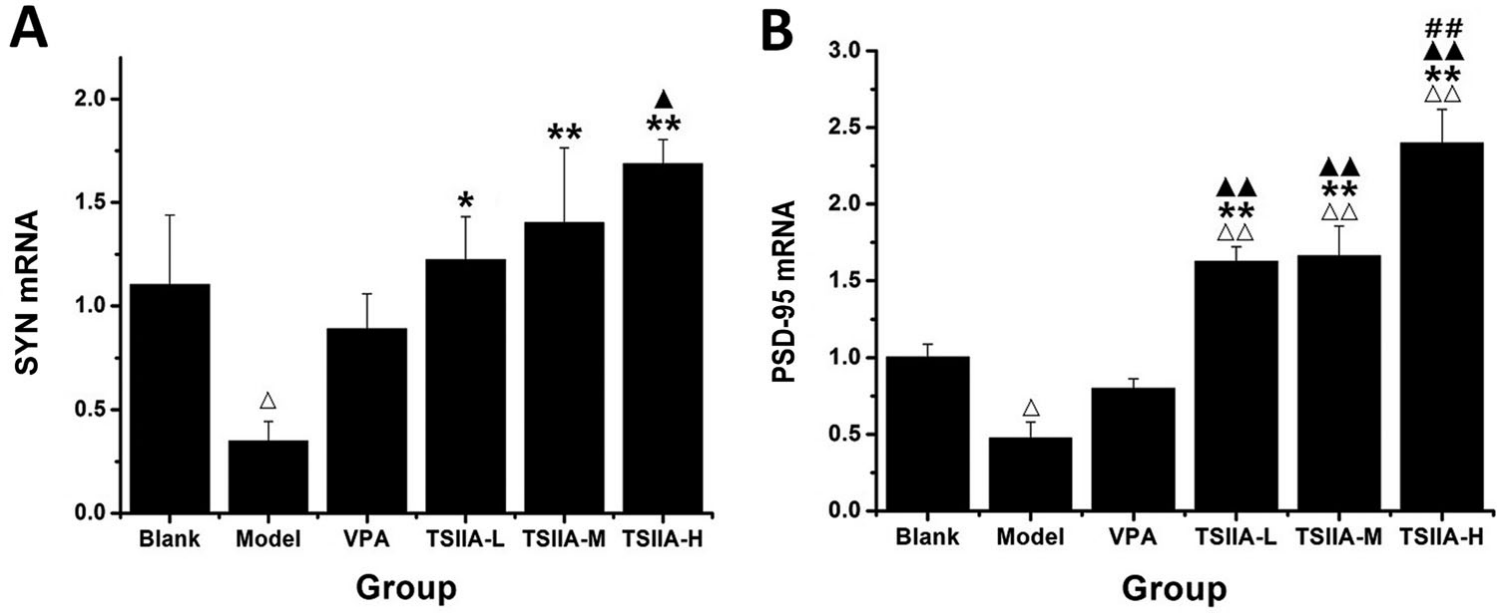
Effect of tanshinone IIA on SYN mRNA and PSD-95 mRNA expression in the hippocampi. (A) Relative expression results of SYN mRNA. (B) Relative expression results of PSD-95 mRNA. The results are presented as means ± SEM. ^Δ^*p* < 0.05, ^ΔΔ^*p* < 0.01 vs. control; *p < 0.05, **p < 0.01 vs. model; ^▲^*p* < 0.05, ^▲▲^*p* < 0.01 vs. VPA; ^##^*p* < 0.01 vs. TS IIA-L (*n* = 3 per group).

In Figure 7 and Table 3, it is evident that a marked decrease in mRNA expression of SYN and PSD-95 for the model and VPA groups was observed compared to the control group (SYN: model 0.35 ± 0.03, VPA 0.53 ± 0.13 vs. control 1.10 ± 0.06; PSD-95: model 0.41 ± 0.08, VPA 0.56 ± 0.04 vs. control 1.00 ± 0.12). The expression of SYN mRNA was also markedly higher in all tanshinone groups (TS IIA-L 0.84 ± 0.03, TS IIA-M 0.98 ± 0.06, TS IIA-H 1.04 ± 0.07 vs. model 0.35 ± 0.03; Figure 7(A)). In contrast to the model group, all tanshinone groups showed significantly increased PSD-95 mRNA expression (TS IIA-L 0.78 ± 0.12, TS IIA-M 0.89 ± 0.12, TS IIA-H 1.02 ± 0.09 vs. model 0.41 ± 0.08), while the VPA group had no apparent change (Figure 7(B)). SYN and PSD-95 mRNA expression was significantly higher in the TSIIA-M and TSIIA-H groups than in the VPA group. As a result, both mRNA and protein expression yielded comparable results.

**Table 3.** Relative expression of SYN and PSD-95 mRNA in the hippocampi of each group.

Group	SYN (Ct)	β-actin (Ct)	SYN mRNA (2^–ΔΔCt^)	PSD-95 (Ct)	β-actin (Ct)	PSD-95 mRNA (2^–ΔΔCt^)
Blank	23.32 ± 0.79	18.57 ± 0.25	1.10 ± 0.06	26.69 ± 0.18	18.64 ± 0.13	1.00 ± 0.12
Model	25.08 ± 0.90	18.82 ± 0.85	0.35 ± 0.03^ΔΔ^	27.69 ± 0.10	18.33 ± 0.41	0.41 ± 0.08^ΔΔ^
VPA	24.13 ± 0.52	18.44 ± 0.40	0.53 ± 0.13^Δ^	27.38 ± 0.27	18.50 ± 0.30	0.56 ± 0.04^ΔΔ^
TSIIA-L	23.23 ± 0.74	18.22 ± 0.38	0.84 ± 0.03*	26.84 ± 0.33	18.43 ± 0.47	0.78 ± 0.12^**,▲^
TSIIA-M	22.93 ± 0.79	18.14 ± 0.18	0.98 ± 0.06^**,▲^	26.53 ± 0.32	18.30 ± 0.37	0.89 ± 0.12^**,▲▲^
TSIIA-H	23.13 ± 0.86	18.43 ± 0.38	1.04 ± 0.07^**,▲^	26.28 ± 0.16	18.26 ± 0.09	1.02 ± 0.09^**,▲▲,#^

Results are presented as mean ± SEM.

^Δ^*p* < 0.05, ^ΔΔ^*p* < 0.01 vs. blank; *p < 0.05, **p < 0.01 vs. model; ^▲^*p* < 0.05, ^▲▲^*p* < 0.01 vs. VPA; ^#^*p* < 0.05 vs. TS IIA-L. (*n* = 3 per group).

## Discussion

The epilepsy model established by pilocarpine more closely resembles the features of human temporal lobe epilepsy (TLE) than maximal electroshock (MES) and pentylenetetrazole (PTZ) models (Löscher 2017; Campos et al. 2018; Arshad and Naegele 2020). After successful modelling, rats have repeated spontaneous seizures and exhibit clinical and pathological features similar to human epilepsy (Wang et al. 2016).

Tanshinone IIA is widely used in the clinic to treat cardiovascular diseases, hyperlipidaemia, cancer and liver diseases (Shi et al. 2019; Yu et al. 2020; Zhou et al. 2021; Zhang et al. 2022). It is also a promising therapeutic candidate for neurological diseases (Subedi and Gaire 2021). According to an increasing number of experimental studies and clinical applications, tanshinone IIA has shown advantageous neuroprotective effects against ischemic stroke, AD and Parkinson’s disease (Jing 2016; Ji et al. 2017; Maione 2018; Subedi and Gaire 2021). In recent years, tanshinone IIA was found to have significant anticonvulsant effects in studies on PTZ-induced epilepsy models in zebrafish and mice (Buenafe et al. 2013). According to research, tanshinone II has a neuroprotective effect in cerebral ischemia by inhibiting the TLR4/NF-B signalling network or by encouraging axonal regeneration by inhibiting the Nogo-A/NgR1/RhoA/ROCKII/MLC signalling system (Fang et al. 2018; Wang et al. 2020). In addition, via stimulating the ERK-CREB-BDNF signalling pathway, tanshinone IIA demonstrates neuroprotective effects in mice with depressive-like behaviour (Lu et al. 2020). Tanshinone IIA's neuroprotective effect may be connected to its anticonvulsant action. Tanshinone IIA also ameliorates lead-induced cognitive impairment in young rats by enhancing antioxidant activity in the brain (Tang et al. 2017). Despite these findings, the mechanisms of its anticonvulsant activities and cognition-protective actions in epileptic mice remain largely unknown. This study established an epilepsy model in rats by injecting LiCl–pilocarpine intraperitoneally. Prolonged seizures in rats reduced their memory and learning ability, but tanshinone IIA displayed anticonvulsant properties and cognitive protection. The behavioural observation indicated that tanshinone IIA treatment effectively suppresses the severity and frequency of epileptic seizures. The escape latency was significantly reduced in all groups of tanshinone IIA when tested in the place navigation test. In the spatial probe trials, the crossing frequency of the platform was increased after tanshinone IIA treatment, all of which supported that tanshinone IIA could reduce epileptogenesis and improve learning and memory.

Synaptic plasticity refers to neurons’ ability to alter synaptic connectivity over time (Shefa et al. 2018; Yepes 2020). The loss of synaptic connections in the hippocampus has been linked to the cognitive disorder in epilepsy, suggesting a vital role in its pathogenesis (Jiang et al. 2015; Tang et al. 2017). The dentate gyrus exhibits aberrant synaptic plasticity associated with MFS in chronic human epilepsy and epileptic animal model (Scharfman et al. 2003; Mello and Longo 2009; Twible et al. 2021). Epilepsy may cause an extensive neuronal loss in the hippocampus (Schoene-Bake et al. 2014; Zhao et al. 2020), followed by neuronal network remodelling characterized by severe MFS and granular cell neurogenesis (Lynch and Sutula 2000; Williams et al. 2002; Sloviter et al. 2006). Numerous researchers believe that the death of hippocampal neurons is a crucial factor in the onset of MFS (Sutula and Dudek 2007). This research demonstrated no apparent structural damage in the hippocampal CA3 regions in any tanshinone IIA treatment group, especially in the TS IIA-M and TS IIA-H groups. In contrast, the VPA and model groups showed obvious abnormal MFS, ultrastructural disorder and vacuolar degeneration. The disorganized ultrastructure and blurred tissue morphology of the CA3 area were improved after tanshinone IIA treatment. Tanshinone IIA administration may assist preserve the normal synaptic connection between neurons and alleviate the ultrastructural abnormality and vacuolar degeneration of the hippocampus CA3 region induced by epilepsy.

Synaptic plasticity is crucial for maintaining normal neuronal function in the nervous system and synaptic repair after cognitive dysfunction (Shefa et al. 2018). The levels of proteins involved in synaptic plasticity alter during epileptogenesis (Royero et al. 2020). Synaptic plasticity-related proteins, especially SYN and PSD-95 proteins, are two typical signature proteins of synaptic remodelling that can directly or indirectly reflect changes in synaptic function and structure (Luo et al. 2013; Guarnieri et al. 2017; Jeong et al. 2019). The presynaptic protein SYN is a glycoprotein in the synaptic vesicle membrane that regulates neurotransmitter release and the differentiation and growth of neuronal protrusions (Mirza and Zahid 2018; Liu et al. 2019). PSD-95 has been utilized as a marker for synaptogenesis and synapse loss, though its specific role is unknown. PSD-95 is a crucial protein in maintaining the stability of synaptic backbone structure and regulating synaptic plasticity in mature glutamatergic synapses (Chen et al. 2011; Jeong et al. 2019). The levels of PSD-95 were reported to be downregulated during epileptogenesis, which may be related to neuronal death or dendritic spine loss in the hippocampus (Jiang et al. 2007; Sun et al. 2009; Frasca et al. 2011). According to our research, the immunofluorescence staining, western blotting and RT-qPCR were consistent at the protein and gene levels. Tanshinone IIA treatment groups all revealed significant increases in SYN and PSD-95 protein and mRNA expression compared to the control group. Tanshinone IIA may have a significant role in regulating synaptic plasticity, implying that the pharmacological mechanism of tanshinone IIA in cognitive disorders of epilepsy may be connected to enhanced PSD-95 and SYN expression and improved synaptic plasticity in the hippocampus.

Based on the Meeh-Rubner formula by conversion of body surface area and the relevant literature (Gouma et al. 2012; He et al. 2020), we selected daily doses of 10, 20 and 30 mg/kg/d tanshinone IIA as low, medium and high doses, respectively. All tanshinone IIA groups showed positive anticonvulsant and cognition-protective effects that were dose-dependent. Previous research reported that high doses of tanshinone IIA in cellular and embryonic models might have cytotoxic effects (Shi et al. 2012; Wang et al. 2017; Yang et al. 2019). The benefits of low doses of tanshinone IIA remain undetermined.

## Conclusions

Tanshinone IIA could effectively reduce epileptogenesis, protect against cognitive impairment, alleviate the abnormal germination of mossy fibres and improve the ultrastructural disorder of the hippocampal CA3 regions. The protective mechanism may be related to the upregulation of the expression level of PSD-95 and SYN proteins and the regulation of synaptic plasticity in LiCl–pilocarpine-induced epileptic rats. New ideas and a theoretical foundation for further research on new clinical indications for tanshinone IIA will be provided by the findings of this study. They will help improve cognitive function in epileptic patients.
